# Minichromosome maintenance protein 10 (*mcm10*) regulates hematopoietic stem cell emergence in the zebrafish embryo

**DOI:** 10.1016/j.stemcr.2023.05.022

**Published:** 2023-07-11

**Authors:** Pietro Cacialli, Serkan Dogan, Tanja Linnerz, Corentin Pasche, Julien Y. Bertrand

**Affiliations:** 1 University of Geneva, Faculty of Medicine, Department of Pathology and Immunology, Rue Michel-Servet 1, 1211 Geneva 4, Switzerland; 2 McMaster University, Faculty of Sciences, Department of Biology, 1280 Main Street West, Hamilton, ON L8S 4K1, Canada; 3 University of Auckland, Faculty of Medical and Health Sciences, Department of Molecular Medicine and Pathology, 85 Park Road, 1023 Auckland, New Zealand; 4 Geneva Centre for Inflammation Research, Faculty of Medicine, University of Geneva, Geneva, Switzerland

**Keywords:** mcm10, hemogenic endothelium, cell cycle, p53, apoptosis, zebrafish, hematopoietic stem cells

## Abstract

Hematopoietic stem cells (HSCs) guarantee the continuous supply of all blood lineages during life. In response to stress, HSCs are capable of extensive proliferative expansion, whereas in steady state, HSCs largely remain in a quiescent state to prevent their exhaustion. DNA replication is a very complex process, where many factors need to exert their functions in a perfectly concerted manner. Mini-chromosome-maintenance protein 10 (*Mcm10*) is an important replication factor, required for proper assembly of the eukaryotic replication fork. In this report, we use zebrafish to study the role of *mcm10* during embryonic development, and we show that *mcm10* specifically regulates HSC emergence from the hemogenic endothelium. We demonstrate that *mcm10*-deficient embryos present an accumulation of DNA damages in nascent HSCs, inducing their apoptosis. This phenotype can be rescued by knocking down *p53*. Taken all together, our results show that *mcm10* plays an important role in the emergence of definitive hematopoiesis.

## Highlights

• *Mcm10* regulates HSC specification from the hemogenic endothelium
• *Mcm10* ensures the stability of the genome in the proliferating hemogenic endothelium
• Nascent HSCs undergo apoptosis in *mcm10*-deficient embryos

## Teaser

In this study, Bertrand and colleagues show that *Mcm10* is necessary to the development of hematopoietic progenitors during the endothelial-to-hematopoiesis process, by guaranteeing the integrity of the genome. Indeed, the absence of *mcm10* results in the accumulation of DNA breaks in the hemogenic endothelium, leading to premature *p53*-dependent apoptosis, suggesting that cell cycle progression is intimately linked to HSC emergence.

## Introduction

In all vertebrates, hematopoietic stem cell (HSC) emergence from the aortic hemogenic endothelium represents a highly conserved process involving an endothelial-to-hematopoietic transition (Bertrand et al., 2010; Kissa and Herbomel, 2010). The specification of the hemogenic endothelium requires many intrinsic and extrinsic signals and leads to the birth of a limited number of HSCs that must mature and expand (Clements and Traver, 2013; North et al., 2007). After a period of expansion during embryonic/fetal life (Mahony and Bertrand, 2019; Paik and Zon, 2010), HSCs will become quiescent during adulthood in the adult bone marrow (adult hematopoietic niche in mammal) and in the zebrafish kidney (Kopp et al., 2005; Song et al., 2004; Stachura et al., 2009; Zhao and Li, 2016). Multiple studies have shown that HSCs enter the cell cycle rapidly after their emergence from the hemogenic endothelium (Fadlullah et al., 2022; Grainger et al., 2016; Porcheri et al., 2020; Zape et al., 2017), before they have reached the fetal liver in mammals or the caudal hematopoietic tissue (CHT) in the zebrafish embryo.

In all eukaryotes, DNA replication always begins with the recruitment of the pre-replication complex (pre-RC), which consists of the origin recognition complex, cell division cycle 6 (Cdc6) protein, Cdc10-dependent transcript 1 (Cdt1), and finally the loading of the replicative helicase’s catalytic core, which is composed of the mini-chromosome-maintenance complex (Mcm) proteins 2–7 (*Mcm2*–*7*) (Kearsey et al., 1996; Liang and Stillman, 1997; Swiech et al., 2007). This is achieved during late mitosis and G1-phase. The additional recruitment of co-factors cell division cycle 45 (Cdc45) and go-ichi-ni-san (GINS) forms the CMG helicase (Aparicio et al., 2006; Gambus et al., 2006; Ilves et al., 2010). Mcm10 participates in this activation process and remains physically attached to the *Mcm2*–*7* complex throughout DNA replication (Mayle et al., 2019; Watase et al., 2012).

Previous functional studies in mice showed that decreased levels of just one component of the *Mcm2*–*7* complex, which are normally present in excess in young and adult HSCs, activated the canonical DNA damage response, inducing high levels of replication-associated γH2AX foci, increased cell cycle defects, altered DNA fork replication dynamics, and chromosome gaps/breaks (Flach et al., 2014).

As mentioned before, while the role of the *Mcm2*–*7* complex in hematopoiesis has been widely described, not much has been described in literature about *Mcm10* in the context of hematopoiesis. Recent *in vitro* studies using human cell lines showed that *MCM10* is critical for human telomere replication and suggest that defective telomere maintenance caused both *MCM10*-associated natural killer (NK) cell deficiency and restrictive cardiomyopathy with hypoplasia of the spleen and thymus (Baxley et al., 2021; Mace et al., 2020). However further investigations need to decipher its role in hematopoiesis.

In the present *in vivo* study, we use zebrafish to investigate the role of *mcm10* in hematopoiesis during embryonic development. We show that *mcm10* regulates HSCs emergence at the level of the hemogenic endothelium, and we demonstrate that *mcm10*-deficient embryos present an accumulation of DNA damages in newly generated HSCs, inducing their apoptosis in a p53-dependent manner. Our results show that *mcm10* is crucial during the emergence of definitive hematopoiesis.

## Results

### *Mcm10* is specifically expressed in HSCs

**Figure 1:**
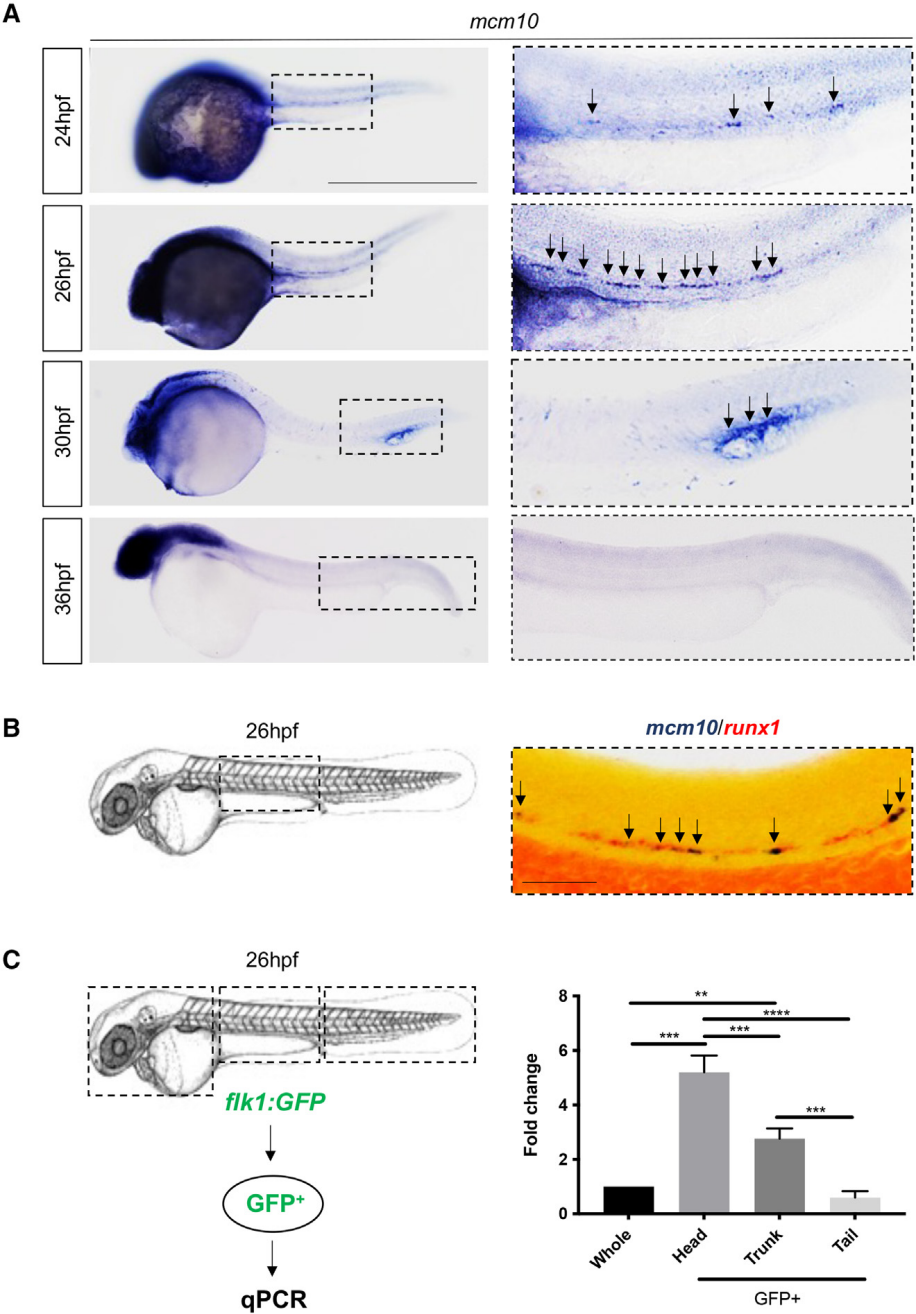
*mcm10* is expressed in the hemogenic endothelium (A) WISH for *mcm10* at different stages of zebrafish embryonic development (24–36 hpf). (B) Double WISH for *mcm10/runx1* at 26 hpf. (C) Experimental outline of qPCR analysis after dissection of heads, trunks, and tails from 26 hpf *flk1:GFP* transgenic animals (around 50 embryos), comparing with whole embryos. Data represent biological triplicates plated in technical duplicates. Statistical analysis was completed using one-way ANOVA, multiple comparison test. ^∗∗^p < 0.01; ^∗∗∗^p < 0.001; ^∗∗∗∗^p < 0.0001. Scale bars: 200 μm (A), 100 μm (B).

In order to investigate the role of *mcm10* in zebrafish developmental hematopoiesis, we first examined its expression pattern using whole-mount *in situ* hybridization (WISH). We found that *mcm10* is initially ubiquitously expressed at 4–8 hours post fertilization (hpf). Its expression is broadly maintained in the following stages of development at 12 and 16 hpf (Figure S1A). Between 24 and 26 hpf, *mcm10* is expressed along the dorsal aorta, as well as in the whole head (Figure 1A). By performing double WISH, we found that *mcm10* is specifically expressed in the hemogenic endothelium, identified with the *runx1* probe (Figure 1B). Further analysis at 26 hpf showed that *mcm10* was enriched in sorted endothelial cells from dissected *flk1:GFP* embryos (Figures 1C and S1B–S1D for gating and sorting strategy) and particularly in GFP^+^ cells sorted from head and trunk regions. These data show a potential link between *mcm10* and embryonic definitive hematopoiesis.

### *Mcm10* deficiency affects HSC emergence in the dorsal aorta

**Figure 2:**
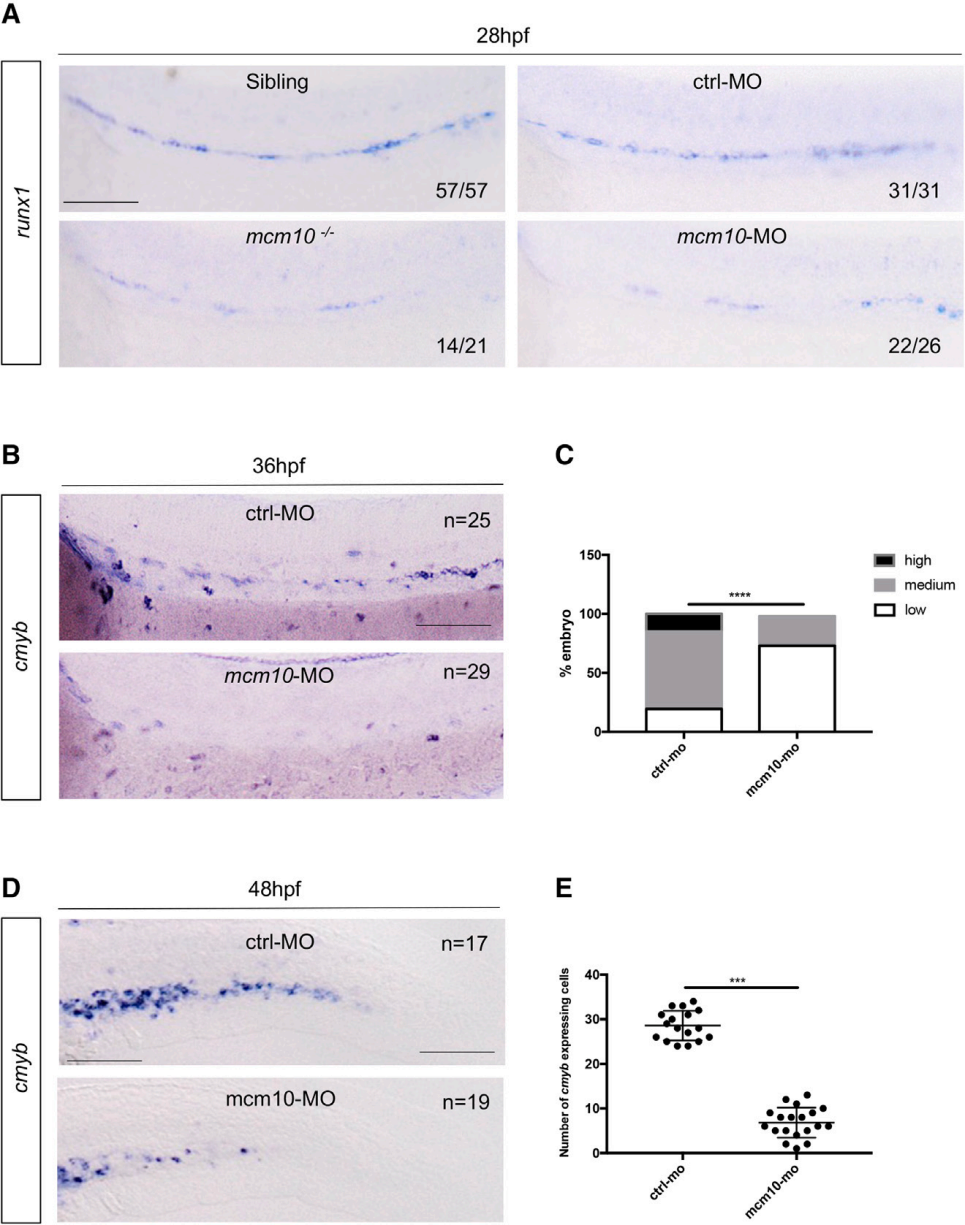
The loss of *mcm10* affects HSCs emergence (A) WISH for *runx1* expression at 28 hpf in *mcm10*^*−/−*^ embryos and their siblings and after *mcm10* knockdown by morpholino injection. (B) WISH for *cmyb* expression along the aorta at 36 hpf in control and *mcm10*-morphants. (C) Statistical analysis was completed using Fisher’s exact test, ^∗∗∗∗^p < 0.0001 (n = number of total embryos from three independent experiments). (D) WISH for *cmyb* expression in the CHT at 48 hpf in control and *mcm10* morphants. (E) Statistical analysis was completed using unpaired two-tailed t test, ^∗∗∗^p < 0.001 (n = number of total embryos from three independent experiments). Scale bars: 100 μm (A, B, and D).

**Figure 3:**
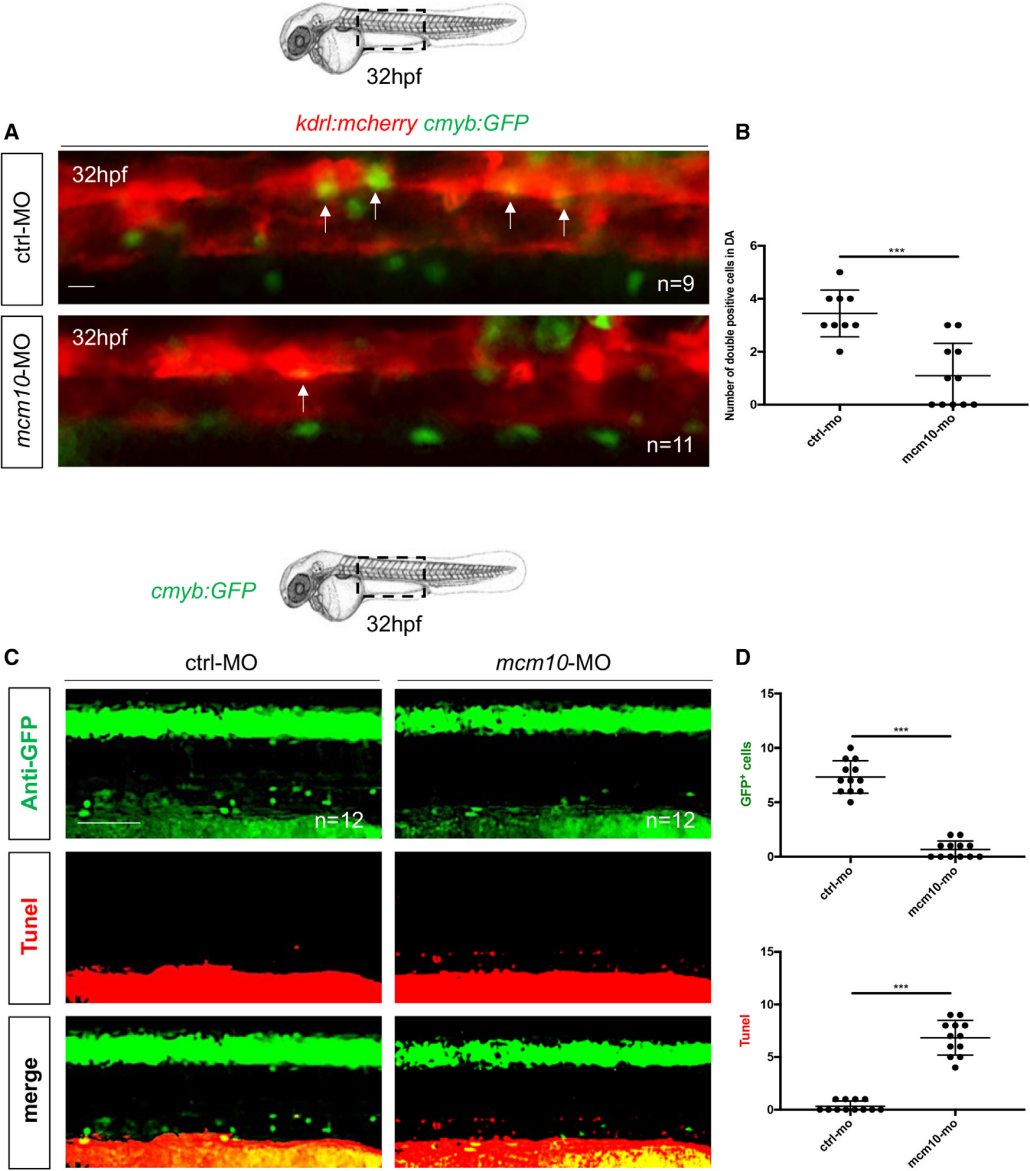
*mcm10* deficiency induces apoptosis in developing HSCs (A) Fluorescence imaging of the dorsal aorta in *flk1:mCherry;cmyb:GFP* embryos injected with control or *mcm10*-MOs. (B) Quantification of HSCs. Statistical analysis: unpaired two-tailed t test, ^∗∗∗^p < 0.001 (n = number of total embryos from three independent experiments). (C) Anti-GFP and TUNEL stainings of *cmyb:GFP* embryos at 32 hpf, after injection of either control or *mcm10* morpholinos *(mcm10*-MO). (D) Quantification of the number of GFP+ and TUNEL+ cells in control and *mcm10*-morphants. Center values denote the mean, error values denote SEM, and statistical analysis was completed using an unpaired two-tailed t test. ^∗∗∗^p < 0.001 (n = number of total embryos from three independent experiments). Scale bars: 50 μm (A), 100 μm (C).

To investigate the role of *mcm10* during developmental hematopoiesis, we examined the consequences of *mcm10* deficiency on HSC development. The uncharacterized *mcm10*^*sa16502*^ mutant line contains a point mutation, which induces a premature stop in exon 8 (Figures S2A–S2C). In these *mcm10*^*−/−*^ mutant embryos, *mcm10* mRNA was undetectable (Figure S2D), indicating that this mutation induces a null allele. By WISH, we found that *mcm10*-deficient embryos present a defect in definitive hematopoiesis, as *runx1* expression was decreased in *mcm10*^*−/−*^ mutant embryos at 28 hpf (Figure 2A, left panels), compared with their siblings, whether wild-type or heterozygous for the mutation. This was consistent with a loss of *cmyb*, observed at 5 days post fertilization (dpf) in the CHT (Figure S3A). Consistently, *mcm10*^−/−^ embryos also showed a decrease of *rag1* expression in the thymus (Figures S3B and S3C). We observed no change in *gata1*, *pu.1*, or *flk1* expression, confirming that *mcm10* is not involved in primitive hematopoiesis nor vasculogenesis (Figures S4A–S4C). We also examined *gata2b* expression, which is an earlier marker than *runx1* for the hemogenic endothelium (Butko et al., 2015) and found no difference in *mcm10*^*−/−*^ mutants (Figure S4D), showing that *mcm10* was not involved in the specification of the hemogenic endothelium but rather in its maturation into HSCs. Morpholino-mediated knockdown of *mcm10* targeting exon 5 induced exon 5 skipping (Figure S2E). These morphants phenocopied *mcm10*^−/−^ mutant embryos, as they exhibited a significant decrease in *runx1* and *cmyb* expression along the aortic floor at 28 hpf (Figure 2A right panel) and 36 hpf (Figures 2B and 2C), respectively. The loss of HSCs, as scored by *cmyb*, was also observable as early as 48 hpf (Figures 2D and 2E) and at 5 dpf (Figure S3D) in the CHT. As a consequence, *mcm10* morphants also showed a decrease of *rag1* expression at 5dpf (Figures S3E and S3F). As in mutants, *mcm10* morphants did not show any phenotype involving primitive erythropoiesis and myelopoiesis, or vasculogenesis, as we found no change in the expression of *pu.1*, *mfap4, mpx*, *gata1*, *flk1*, or *efnb2a* (arterial marker) as well as *gata2b* (Figures S5A and S5B). To confirm previous observations, we injected control and *mcm10* morpholinos in *kdrl:mCherry;cmyb:GFP* double-transgenic embryos and counted the number of HSCs emerging from the dorsal aorta at 32 hpf (Figure 3A). *Mcm10* morphants exhibited a significant decrease of double-positive cells compared with controls (Figure 3B). As these results suggested that the *mcm10* deficiency might induce the cell death of HSCs, we performed TUNEL assays in *cmyb:GFP* embryos at 32 hpf. *Mcm10* morphants showed a significant increase of cell death in the dorsal aorta, correlating to the absence of GFP-positive cells (Figures 3C and 3D), suggesting that cell death by apoptosis occurred at early stages of HSC development.

### *Mcm10* overexpression increases HSCs emergence from the hemogenic endothelium

**Figure 4:**
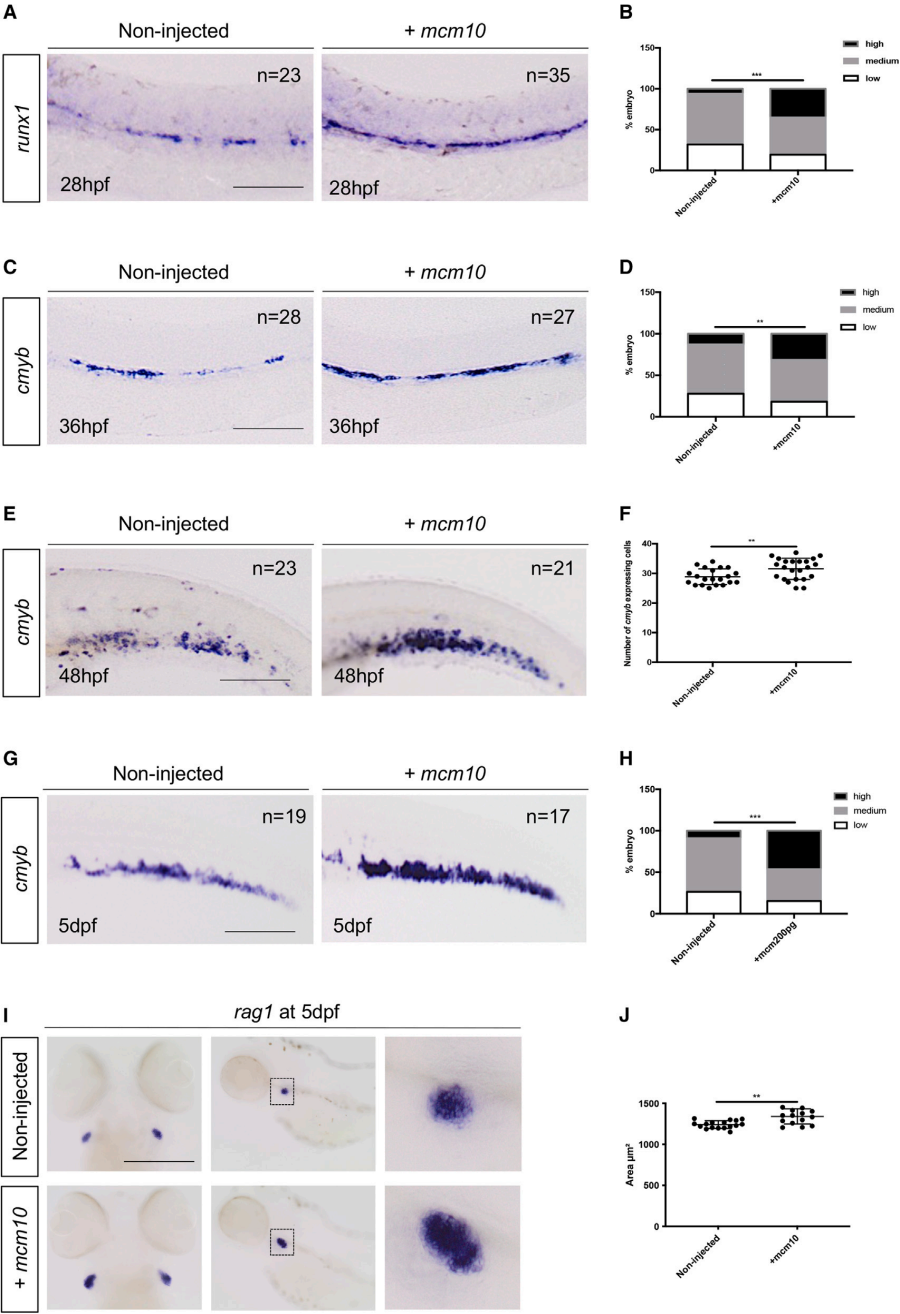
*mcm10* overexpression increases the expression HSC marker (A) *runx1* expression in 28-hpf embryos, either non-injected or injected with *mcm10* full-length mRNA. (B) Statistical analysis was completed using Fisher’s exact test. ^∗∗∗^p < 0.001 (n = number of total embryos from three independent experiments). (C) WISH against *cmyb* at 36 hpf in embryos, either non-injected or injected with *mcm10* full-length mRNA. (D) Statistical analysis was completed using Fisher’s exact test. ^∗∗^p < 0.01 (n = number of total embryos from three independent experiments). (E) *cmyb* expression in 48-hpf embryos, either non-injected or injected with *mcm10* full-length mRNA. (F) Quantification of the number of *cmyb*-positive cells in the CHT per embryo. Statistical analysis was completed using an unpaired two-tailed t test. ^∗∗^p < 0.01 (n = number of total embryos from three independent experiments). (G) WISH against *cmyb* in 5-dpf embryos, either non-injected or injected with *mcm10* full-length mRNA. (H) Statistical analysis was completed using Fishers exact test. ^∗∗∗^p < 0.001. (I) WISH against *rag1* at 5 dpf in embryos, either non-injected or injected with *mcm10* full-length mRNA. (J) The area of the thymus was measured for each embryo. Statistical analysis was completed using an unpaired two-tailed t test. ^∗∗^p < 0.01 (n = number of total embryos from three independent experiments). Scale bars: 100 μm (A, C, E, G, and I).

**Figure 5:**
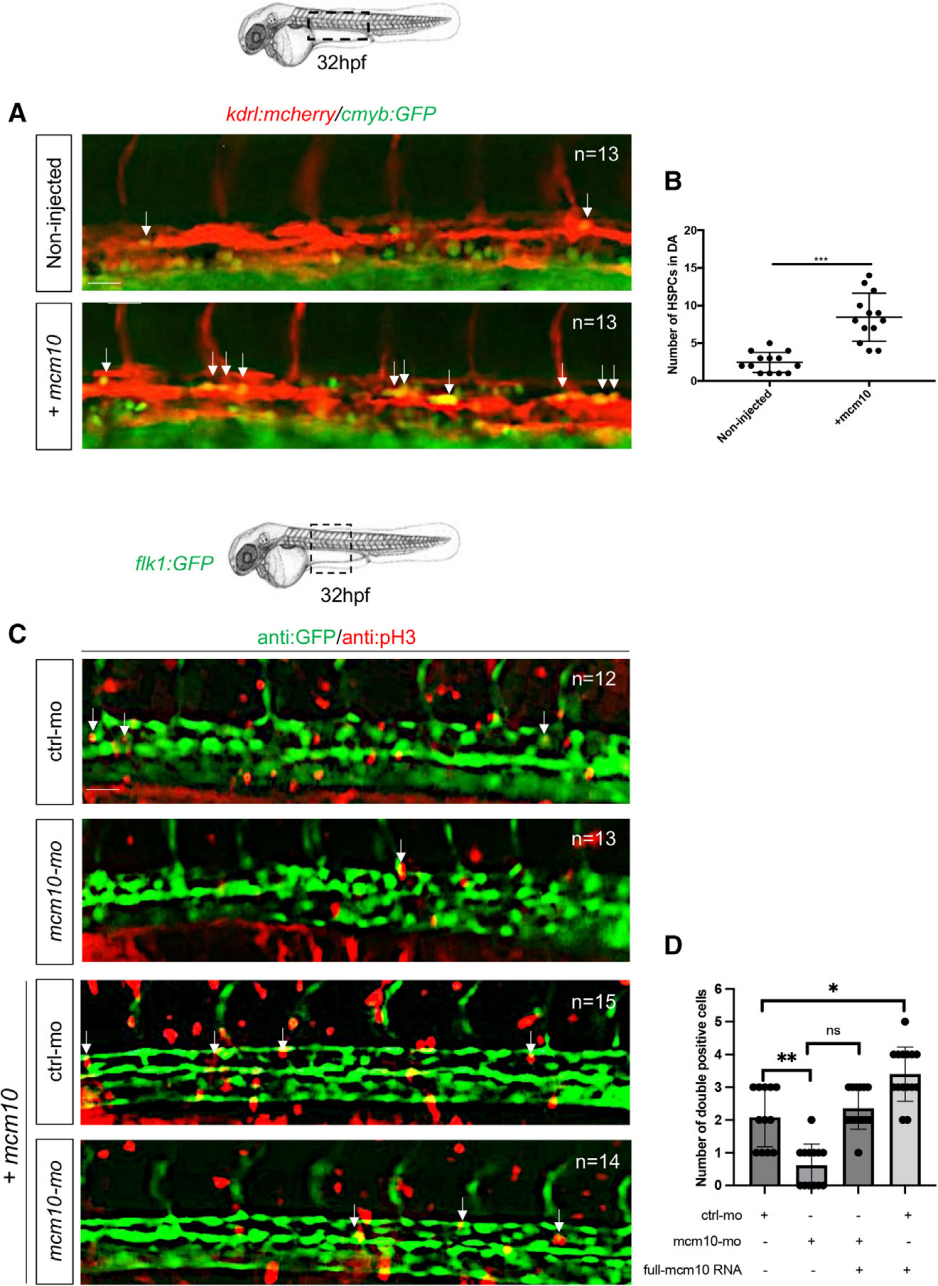
*Mcm10* overexpression increases the proliferation of emerging HSCs at the level of the hemogenic endothelium (A) Fluorescence imaging of dorsal aorta in 32-hpf *kdrl:mcherry/cmyb:GFP* double-transgenic embryos, either non-injected or injected with *mcm10* full-length mRNA. (B) The number of double-positive cells was reported for each condition. Statistical analysis was completed using an unpaired two-tailed t test. ^∗∗∗^p < 0.0001. Center values denote the mean, and error values denote SEM (n = number of total embryos from three independent experiments). (C) An immunofluorescence against GFP and phospho-histone 3 (pH3) was performed on 32-hpf *flk1:GFP* transgenic embryos, either injected with control or *mcm10* morpholinos, and with *mcm10* full-length mRNA. The aorta region was imaged and (D) the number of double-positive cells (representing the proliferating endothelial cells) was scored for all embryos in each condition. Center values denote the mean, error values denote SEM, and statistical analysis was completed using ANOVA multiple t test. ^∗^p < 0.01, ^∗∗^p < 0.001 (n = number of total embryos from three independent experiments). Scale bar: 50 μm (A–C).

We next investigated the effect of *mcm10* overexpression on embryonic hematopoiesis. We induced overexpression of *mcm10* by injecting the full-length mRNA at the one-cell stage and analyzed the effect on developmental hematopoiesis. *Mcm10* overexpression did not change primitive hematopoiesis (as marked by *pu.1* and *gata1*) or blood vessel development (using the *flk1:GFP* transgenic line) at 24 hpf (Figures S6A–S6C). We found that *gata2b* expression was not affected by *mcm10* overexpression (Figure S6D); however, *mcm10* overexpression increased *runx1* and *cmyb* expression, at 28 and 36 hpf, respectively, showing that *mcm10* is critical for the *gata2b-*to-*runx1* transition during HSC development (Figures 4A–4D). This resulted in increased numbers of HSCs at 48 hpf and 4.5 dpf in the CHT (Figures 4E–4H), as marked by *cmyb*. This increase of HSCs was also maintained in the thymus (Figures 4I–4L), as marked by *rag1* staining. To further analyze the increase of *cmyb*^+^ cells, we imaged double-positive *kdrl:mCherry;cmyb:GFP* embryos to examine HSC emergence from the hemogenic endothelium. *Mcm10* overexpression significantly increased the number of double-positive, nascent HSCs in the dorsal aorta at 32 hpf (Figures 5A and 5B). Accordingly, the number of HSCs in the CHT niche at 42 hpf was also significantly increased (Figures S7A and S7B). Indeed, previous functional *in vitro* studies showed that the overexpression of *mcm10* improved progression through the cell cycle, while knockdown by RNA interference reduced cell proliferation (Cui et al., 2018; Kang et al., 2020; Watase et al., 2012). In order to verify this hypothesis in our *in vivo* model, we injected *mcm10* mRNA in *mcm10*-deficient *flk1:GFP* transgenic embryos (injected with *mcm10* morpholino) and performed phospho-histone H3 (pH3) staining to measure proliferation. *Mcm10* overexpression in control morphants indeed increased the number of proliferating cells (Figures 5C and 5D), but more importantly, it could rescue the decrease of cell proliferation observed in *mcm10* morphants (Figures 5C and 5D). Accordingly, *mcm10* overexpression also rescued the decrease of *runx1* expression observed in *mcm10* morphants (Figure S7C). Taken all together, these results show that *mcm10* is involved in HSC emergence during embryonic development, probably by modulating the progression of hemogenic endothelial cells through the cell cycle.

### *Mcm10*-deficient embryos accumulate DNA damages in HSCs

**Figure 6:**
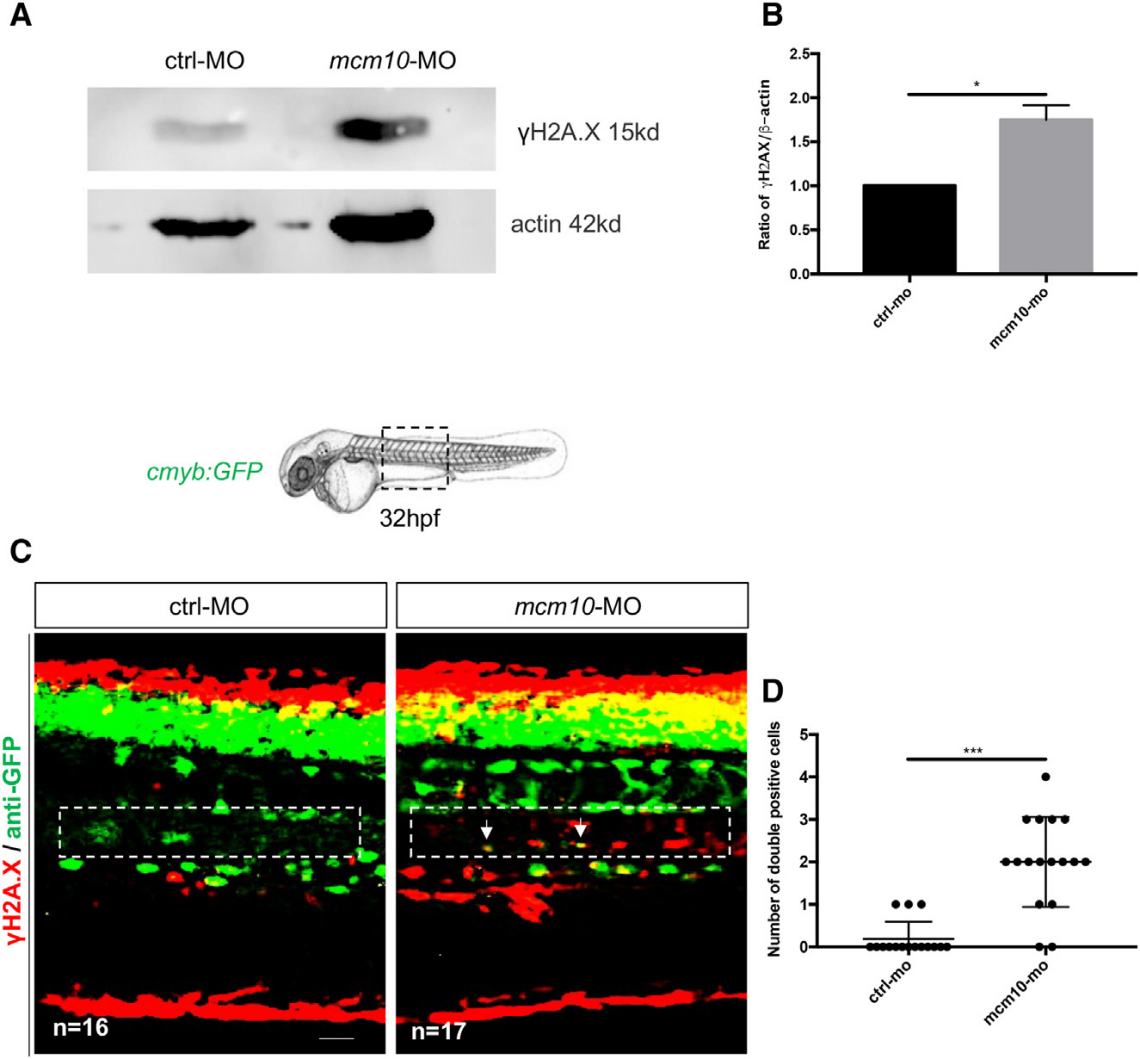
*mcm10*-deficient embryos accumulate DNA damages in nascent HSCs (A) Western blot to quantify γH2A.X in control or *mcm10* morphants. (B) Statistical analysis of the ratio γH2A.X/actin was completed using an unpaired two-tailed t test. ^∗^p < 0.01 (three independent experiments, with >30 embryos pooled per condition, per experiment). (C) Anti-GFP and γH2A.X stainings performed on 32-hpf *cmyb:GFP* embryos, either injected with control or *mcm10* morpholinos. (D) Quantification of the number of double-positive cells in the aorta floor of control and *mcm10* morphants. Center values denote the mean, error values denote SEM, and statistical analysis was completed using an unpaired two-tailed t test. ^∗∗∗^p < 0.001 (n = number of total embryos from three independent experiments). Scale bar: 50 μm (C).

We next set out to decipher the mechanism by which *mcm10* regulates hematopoiesis. Previous functional studies in human cells reported that *Mcm* genes (*Mcm*2–7) are usually present in excess, which protects the genome under replication stress (Ibarra et al., 2008). Flach and colleagues showed that decreased levels of *mcm* genes activated the canonical DNA damage response, inducing high levels of replication-associated γH2A.X foci and increased levels of replication stress associated with cell cycle defects (Flach et al., 2014). It is commonly accepted that γH2A.X foci mark altered DNA fork replication and chromosome gaps/breaks. To verify this hypothesis in our zebrafish model, we first measured γH2A.X protein levels in AB^∗^ embryos injected with control and *mcm10* morpholinos. We found a significant increase of γH2A.X in *mcm10* morphants (Figures 6A and 6B). Next, we evaluated the presence of γH2A.X specifically in HSCs. We injected *cmyb:GFP* transgenic embryos with control and *mcm10* morpholinos and performed γH2A.X immunostaining at 32 hpf. *Mcm10* morphants harbored a significant increase of DNA damages in cells in the aortic floor/hemogenic endothelium, in line with our previous observations (Figures 6C and 6D). This result suggests that *Mcm10* plays an equally important role in sustaining a proper DNA cell cycle progression as has previously been shown for *Mcm2*–*7*.

### The loss of HSCs in *mcm10*-deficient embryos can be rescued by inhibiting *p53*

**Figure 7:**
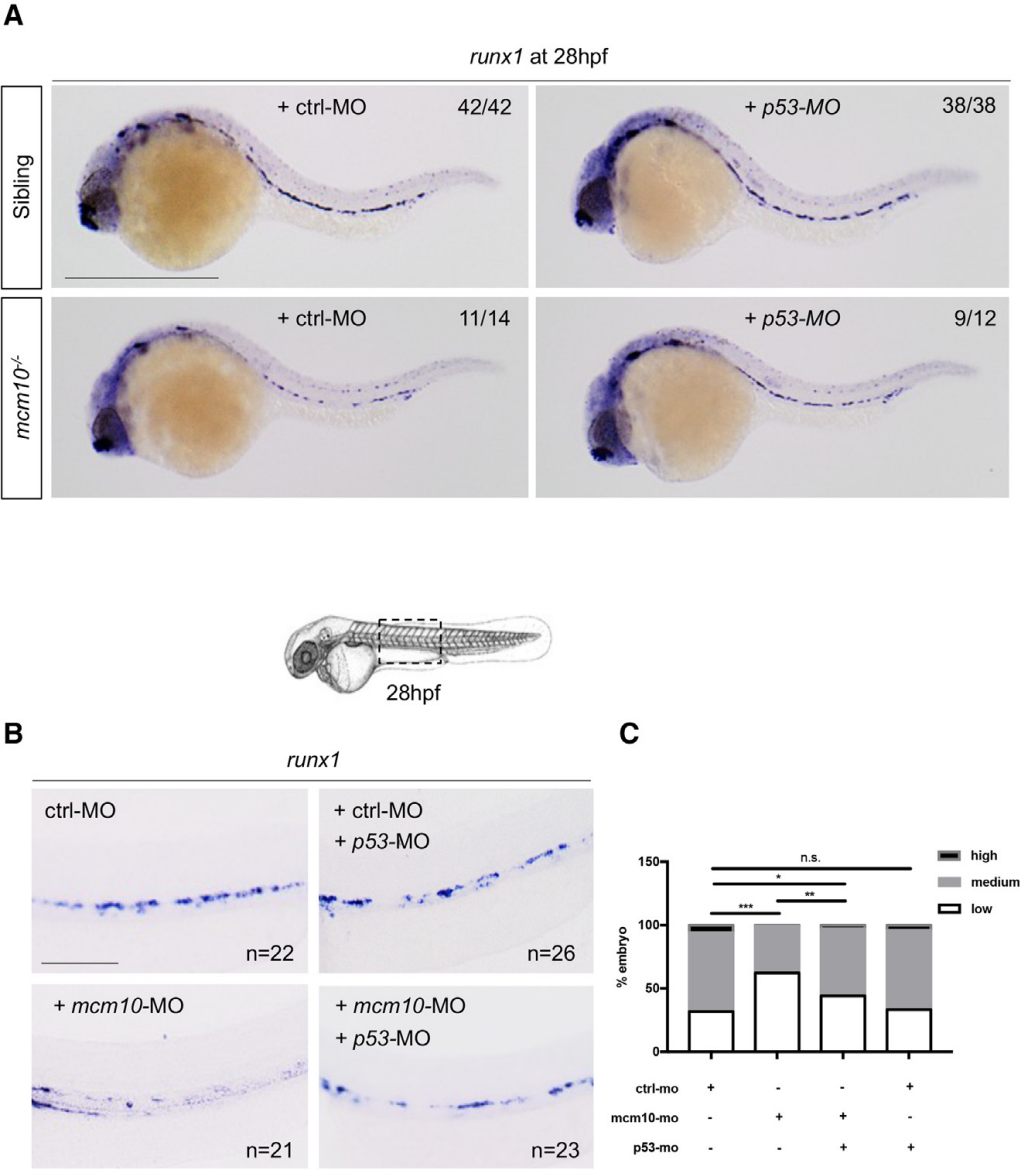
*p53* knockdown rescues the loss of HSCs in *mcm10*-deficient embryos (A) *runx1* expression at 28 hpf in *mcm10*^*−/−*^ mutant embryos and their siblings, either injected with control or *p53* morpholinos. (B) *runx1* expression at 28 hpf in embryos co-injected with *mcm10* and *p53* morpholinos. (C) Statistical analysis was completed using Fisher’s exact test. ^∗^p < 0.01; ^∗∗^p < 0.001; ^∗∗∗^p < 0.0001 (n = number of total embryos from three independent experiments). Scale bars: 200 μm (A), 100 μm (B).

We have shown that *mcm10*-deficient embryos fail to produce HSCs from the hemogenic endothelium. Indeed, the absence of *mcm10* appears to block cell cycle at early stages of HSC specification, inducing apoptosis. In order to inhibit apoptosis in the hemogenic endothelium from *mcm10-*deficient embryos, we co-injected control or *p53* morpholinos in siblings and *mcm10*^*−/−*^ mutant embryos and analyzed *runx1* expression at 28 hpf. We found that *runx1* expression was significantly rescued in *mcm10*^*−/−*^ mutants injected with the *p53* morpholino (Figure 7A). We obtained similar results in *mcm10* morphants (Figures 7B and 7C). In conclusion, *mcm10* is necessary for the development of aortic HSCs, as its absence results in *p53*-dependent apoptosis of hemogenic endothelial precursors at the *gata2b*-to-*runx1* transition.

## Discussion

In this study, we used the zebrafish model to investigate the role of *mcm10* during embryonic development. We show that *mcm10* specifically regulates HSC emergence from the hemogenic endothelium. We found that *mcm10*-deficient embryos present an accumulation of DNA damages in nascent HSCs, resulting in their apoptosis. Accordingly, this phenotype can be rescued by knocking down *p53*. These results are in line with previous published studies, reinforcing the hypothesis that the replication factor *mcm10* plays a pivotal role in cell cycle regulation of HSCs—and potentially in other stem cells—during both fetal and adult life (Flach et al., 2014). The cell cycle activity of HSCs is carefully modulated by a complex interplay between cell-intrinsic mechanisms and cell-extrinsic factors produced by the microenvironment. It has been widely reported that during fetal life, the primordial function of HSCs is to rapidly generate homeostatic levels of blood cells for oxygen transport and to establish the immune system in the developing organism. In line with this role, between 95% and 100% of HSCs are actively cycling in the mouse fetal liver (E14 stage) with a cell cycle transit time ranging from 10 to 14 h (Pietras et al., 2011). Remarkably, murine adult bone marrow HSCs rapidly switch to a quiescent state by 4 weeks of age, as only 5% of total HSCs are actively engaged in the cell cycle (Bowie et al., 2006). The transition from active cell cycling in fetal HSCs to quiescence in adult HSCs could be associated with changes in gene expression programs. As mentioned before, recent studies in mice reported that all members of the *Mcm* gene family are involved in the regulation of the cell cycle in HSCs. *Mcm2*—*7* genes are normally overrepresented in human cells, which allows us to maintain the integrity of the genome under replication stress (Ibarra et al., 2008). We observed a similar increase of DNA damages in cells from the hemogenic endothelium in our *mcm10-*deficient zebrafish model. At the same time, we found that overexpression of *mcm10* increased the expression of the transcription factor *runx1*, reinforcing the hypothesis that *mcm10* plays an important role in the initial steps of HSCs development from the hemogenic endothelium. Our results are in line with previous observations from the drosophila model. Using ChIP assays in S2 cells and quantitative RT-PCR in *Mcm10* knockdown flies, it was shown that *Mcm10* could be involved in the transcriptional regulation of the *lozenge* gene, the *runx1* orthologue in flies (Vo et al., 2014). By immunoprecipitating chromatin with an anti-*Mcm10* antibody, the authors of the study could amplify the upstream region of the *lozenge* gene that contains binding sites for various insulator associated factors such as MDG4, Su(Hw), CTCF, CP190, and BEAF32 (Vo et al., 2014). This set of data suggests that *Mcm10* could also directly regulate *lozenge* and eventually *runx1*, in drosophila and vertebrates, respectively, although the precise mechanism for this regulation would need to be characterized.

The specific phenotype of our *mcm10*-deficient zebrafish model is of particular interest. As a major player in the cell cycle/DNA replication, we would have expected that the *mcm10* deficiency would induce a lethal phenotype at very early stages of embryonic development. Although the embryonic deficiency might be covered by maternally derived RNAs at early stages, this does not explain why this deficiency would first induce a phenotype in the hematopoietic system. However, this has also been observed in neonatal human patients, where mutations in *MCM10* have been associated with specific hematopoietic diseases (Baxley et al., 2021; Mace et al., 2020). Two recent studies have reported that heterozygous mutations in *MCM10* induced natural killer cell deficiency (NKD) or fetal restrictive cardiomyopathy (RCM) with thymic and splenic hypoplasia in children (aged between 16 and 24 months). The NKD-associated variants were identified in a single family and included one missense mutation (c.1276C>T, p.R426C) in exon 10 and one nonsense variant introducing a premature stop codon at the end of exon 13 (c.1744C>T, p.R582X) (Mace et al., 2020). Of note, this NKD was also associated with a severe decrease in the number of other hematopoietic lineages, in particular T and B cells. Concerning RCM, associated variants were identified in three affected siblings and included one splice donor site (c.764+5G>A, p.D198GfsTer10) and one frameshift variant (c.236delG, p.G79EfsTer6) (Baxley et al., 2021). The absence of pathology in family members carrying a single *MCM10* variant demonstrates that mono-allelic null mutations are not haploinsufficient in human beings. Because each heterozygous combination included one null allele, the hypomorphic alleles in each pair seem necessary to further reduce *MCM10* function and elicit disease. Considering this, it could be hypothesized that these specific hematopoietic diseases in *MCM10*-deficient human patients were caused by an alteration of *RUNX1* expression. Indeed, several studies have demonstrated that *RUNX1* drives transcriptional programs that promote NK cell maturation and proliferation (Ohno et al., 2008; Seo et al., 2012). In recent transcriptome analyses, it was suggested that *Runx1*-deficient NK cells from CMV-infected mice display a substantial downregulation of cell cycle and proliferation genes (Rapp et al., 2017). In this context, future transcriptomic and functional studies will be necessary to understand the exact mechanism through which *MCM10* regulates *RUNX1* expression, which could lead to better therapies to treat hematopoietic disorders.

## Experimental procedures

### Resource availability

#### Corresponding author

Julien Y. Bertrand (julien.bertrand@unige.ch).

#### Materials availability

The materials included in the current story are available from the corresponding author on reasonable request.

### Zebrafish husbandry

AB^∗^zebrafish strains, along with transgenic strains and mutant strains, were kept in a 14/10-h light/dark cycle at 28°C. In this study we used the following transgenic lines: *Tg(cmyb:GFP)*^*zf169*^*; Tg(kdrl:Has.HRASmCherry)*^*s896*^*; Tg(kdrl:GFP)*^*s843*^*.* We also used the mutant line *mcm10*^*sa16502*^(purchased from eZRC).

### Identification of *mcm10* mutant line

The mutant line *mcm10*^*sa16502*^ presents the point mutation C/A, which induces a premature stop in exon 8. The *mcm10*^*sa16502*^ line was purchased from the Zebrafish International Resource Center (ZDB-ALT-130411-4969). The *mcm10*^*sa16502*^ line used in this paper was subsequently outcrossed with WT AB^∗^ for clearing of potential background mutations derived from the random ENU mutagenesis (from which this line originated). Genotyping was performed by PCR of the *mcm10* gene followed by sequencing. Primers used for genotyping were as follows: forward GGTTGTTTCTTTGGCTGTGG and reverse GCTCAGCCTGACAGTGGATC.

### Whole-mount *in situ* hybridization and analysis

*cmyb, runx1, pu.1, mfap4, mpx, gata1, gata2b, flk1,* and *rag1* antisense probes (digoxigenin and fluorescein labeled) were previously described and used in our laboratory (Cacialli et al., 2021, 2022; Mahony et al., 2021). WISH was performed on 4% paraformaldehyde-fixed embryos. All injections were repeated three separate times. Analysis was performed using the unpaired Student’s t test, ANOVA multiple comparison test, and/or Fisher’s test (GraphPad Prism). Embryos were imaged in 100% glycerol, using an Olympus MVX10 microscope. Oligonucleotide primers used for the production of the *mcm10* probe were as follows: forward GAGACGGTGCTAAAGTGAAC and reverse CTTCCACAGGTCAGTGTGAA.

### Flow cytometry on zebrafish embryos

Transgenic zebrafish embryos were incubated with a Liberase-Blendzyme3 (Roche) solution for 90 min at 33°C and then dissociated and resuspended in 0.9× PBS-1% fetal calf serum. We excluded dead cells by SYTOX-red (Life Technologies) staining. Cell sorting was performed using an Aria II (BD Biosciences).

### Quantitative real-time PCR on zebrafish cells

Total RNA was extracted using the RNeasy Mini Kit (Qiagen) and reverse transcribed into cDNA using Superscript III (Invitrogen). Quantitative real-time PCR (qPCR) was performed using KAPA SYBR FAST Universal qPCR Kit (KAPA BIOSYSTEMS) and run on a CFX connect real-time system (Bio-Rad).

### Morpholino injections

The *mcm10* morpholino oligonucleotide (MO) and control MO were purchased from GeneTools (Philomath, OR). MO efficiency was tested by reverse transcription polymerase chain reaction (RT-PCR) of total RNA extracted from ∼15 embryos at 28 hpf. In all experiments, 8 ng of *mcm10*-MO was injected per embryo. Morpholinos and primer sequences were as follows: standard control MO CCTCTTACCTCAGTTACAATTTATA, *Mcm10*-MO CAGATATGCTGCTCCATTACATTGT, forward GAGACGGTGCTAAAGTGAAC (exon 4), and reverse CTTCCACAGGTCAGTGTGAA (exon 6).

### Synthesis of full-length mRNA and microinjection

To synthesize the sequence for *mcm10* (full-length mRNA), two PCR products were amplified and subsequently inserted in a pCS2+ vector by In Fusion cloning (Takara). After linearization of the plasmid containing the *mcm10* full-length coding sequence, the mMessage mMachine SP6 kit (Ambion) was used to produce *mcm10* capped mRNA. After transcription, mRNA was purified by phenol chloroform extraction. 200 pg of *Mcm10* mRNA was injected into one-cell-stage embryos. The following primers were designed using the Takara software to amplify the *mcm10* full-length mRNA:

Oligo1-F CTTGTTCTTTTTGCAGGATCCATATTAAACATTTTGCGCGCCA,

Oligo2-R TAGTTGGGATGGATGCCAAAGCAGACTGT,

Oligo3-F TTTGGCATCCATCCCAACTAAGAAACTCGTTCTG, and

Oligo4-R CTATAGTTCTAGAGGCTCGAGTGAAGTTCAAGCAGGTTCAG.

### Confocal microscopy and immunofluorescence staining

Transgenic fluorescent embryos were embedded in 1% agarose dissolved in 1xE3 in a glass-bottom dish. For immunofluorescence, double staining was performed using chicken-*anti*-GFP (1:500; Life Technologies), rabbit-*anti*-H2A.X (phospho Ser139) (1:500; GTX127342; Genetex) rabbit, and pH3 (1:250; Abcam) antibodies. We used AlexaFluor488-conjugated anti-chicken (1:1,000; Life Technologies) and AlexaFluor594-conjugated anti-rabbit (1:1,000; Life Technologies) secondary antibodies to reveal primary antibodies. Confocal imaging was performed using a Nikon inverted A1r spectral.

### TUNEL cell death detection

Cell death was detected by TUNEL assay (*in Situ* Cell Death Detection Kit, Roche). Embryos were fixed overnight with 2% paraformaldehyde at 4°C. After gradual rehydration, the embryos were permeabilized with 25 μg/mL proteinase K for 10 min at 28°C followed by 4% paraformaldehyde (15 min at room temperature) and incubated with 90 μL labeling solution plus 10 μL enzyme solution at 37°C for 2 h. After three 5-min washes in PBT, embryos were imaged by confocal microscopy.

### Western blot analysis

Pools of 50 embryos were collected. Cells were lysed in Pierce IP Lysis Buffer (Thermo Scientific) with protease inhibitor cocktail (Merck), and protein was extracted. Proteins were separated by SDS gel electrophoresis as previously described (Sun et al., 2011) and incubated overnight at 4°C with anti-H2A.X (phospho Ser139) (1:1,000; GTX127342; Genetex) followed by goat anti-rabbit IgG (H1L)-horseradish peroxidase conjugated secondary antibody (1:5,000; 1,706,516; Bio-Rad). Staining was revealed by using Western Bright Sirius (1:1; Adventa) with an exposure of 1 min and 15 s.

## Author contributions

T.L. initiated the project and described the initial phenotype; S.D. established the link between the *mcm10* mutation and the hematopoietic phenotype. S.D. and P.C. performed most of the experiments. C.P. performed western blots and provided technical assistance. P.C. and J.Y.B designed the research and wrote the manuscript.

## Data Availability

All raw data are freely accessible on the following link: https://doi.org/10.26037/yareta:zeuevn4a4vevfhxccx5nag22xm.

## Supplemental information

Document S1. Figures S1–S7

Document S2. Article plus supplemental information
