# Supplementary material for: Minichromosome maintenance protein 10 (mcm10) regulates hematopoietic stem cell emergence in the zebrafish embryo
Source: Stem Cell Reports. 2023 Jul 11;18(7):1534–46. doi: 10.1016/j.stemcr.2023.05.022 (PMC10362509; doi:10.1016/j.stemcr.2023.05.022)
Supplement: Document S1. Figures S1–S7 [file mmc1.pdf]

Stem Cell Reports, Volume *18*

Supplemental Information

Minichromosome maintenance protein 10 (*mcm10*) regulates hematopoietic stem cell emergence in the zebrafish embryo

Pietro Cacialli, Serkan Dogan, Tanja Linnerz, Corentin Pasche, and Julien Y. Bertrand

Supplementary figure and legends

Supplementary figure 1

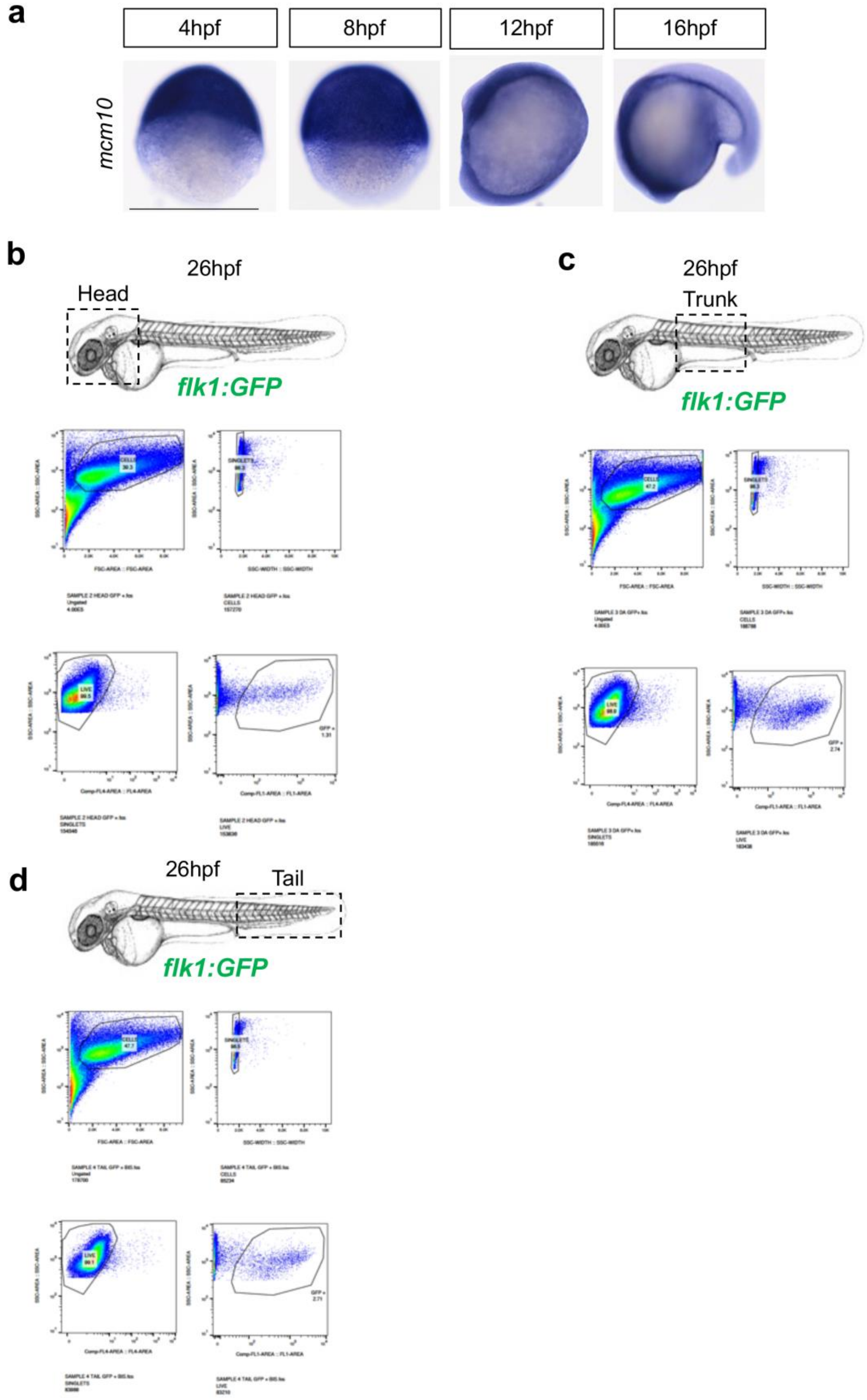

Suppl. Fig.1 *mcm10* pattern expression during early stages of zebrafish embryo, and sorting strategy of endothelial cells at 26hpf. Related to figure 1.

(a) WISH for *mcm10* at different stages (4 to 16hpf) of zebrafish embryonic development. (b-d) Gating strategy to sort GFP+ cells from the heads, trunks and tails of *kdrl:GFP* embryos (three independent sorts were made from >50 pooled embryos). For all samples, cells were gated according to their size, then excluding doublets and dead cells. Scale bar: 200 µm (a).

Supplementary figure 2

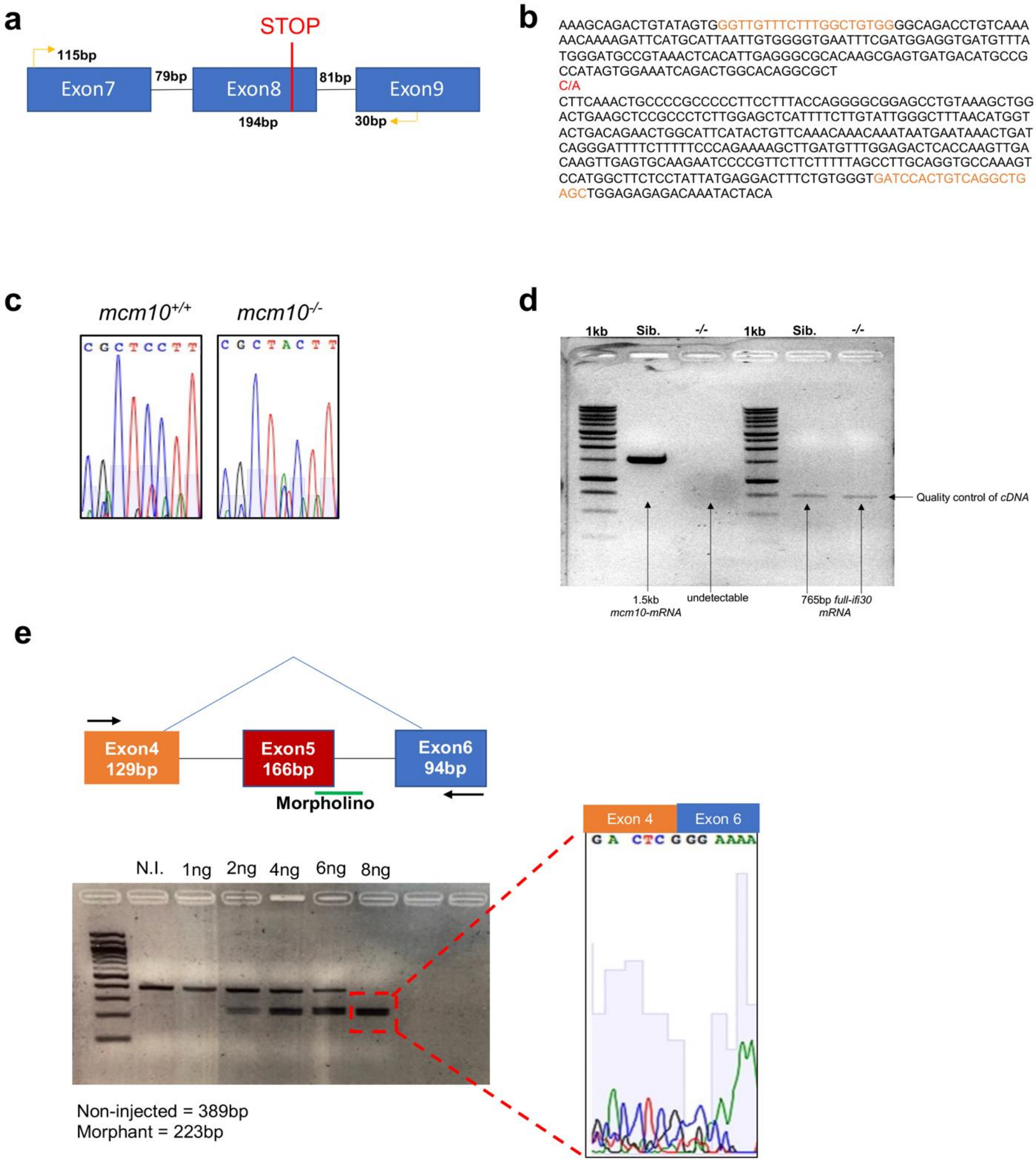

Suppl. Fig.2 Identification of a premature stop in *mcm10*-mutants and morpholino strategy. (a) Schematic outline of the *mcm10* gene with the ENU mutation (orange sequences = primers). (b) Sequence used for genomic PCR during genotyping. (c) Sanger sequencing of PCR product in WT and *mcm10*$^{-/-}$. (d) Agarose gel after amplification of *mcm10-RNA* by RT-PCR (1.5kb oligo1-oligo2 reported in materials and methods) in sibling and *mcm10*$^{-/-}$. We checked the quality of cDNA using primers that amplified *the ifi30 full-length mRNA*. (e) Schematic outline of *Mcm10*-morpholino

strategy (exon 5 skipped), agarose gel of PCR product obtained after mcm10-morpholino injection at different dilution. Sanger sequencing of PCR product.

Supplementary figure 3

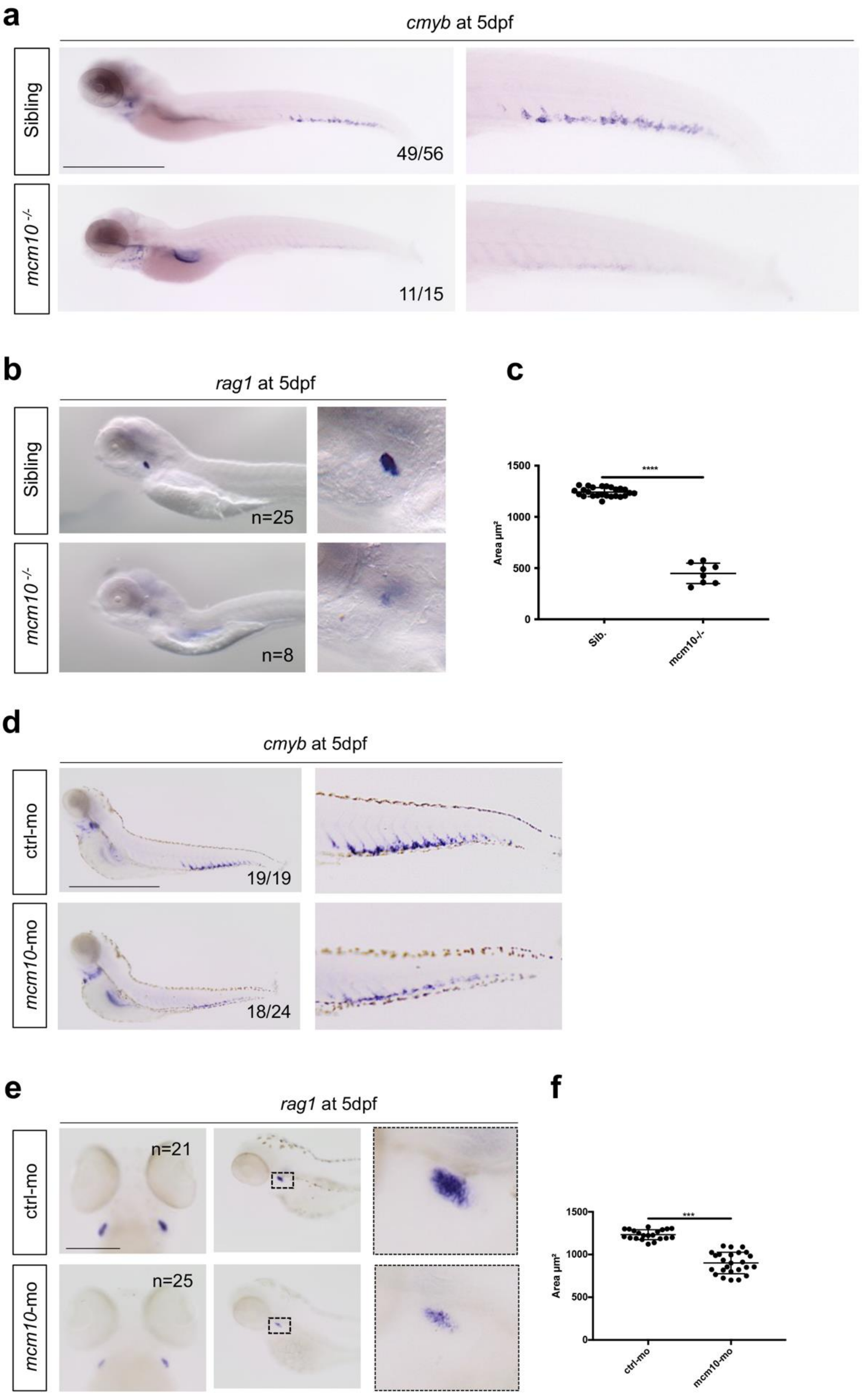

Suppl Fig.3 Mcm10-deficient embryos show the loss of *cmyb* and *rag1* expression at 5dpf. (Related to figure 2).

(a) WISH for *cmyb* at 5dpf in sibling and *mcm10*$^{-/-}$. (b) WISH for *rag1* at 5dpf in sibling and *mcm10*$^{-/-}$. (c) Centre values denote the mean, and error values denote s.e.m, statistical analysis was completed using un-paired two tailed t test, ****P <.0001 (n=number of total embryos from three independent experiments) (d) WISH for *cmyb* at 5 dpf in control and mcm10-morpholino. (e) WISH for *rag1* at 5 dpf in control and mcm10-morpholino. (f) Centre values denote the mean, and error values denote s.e.m, statistical analysis was completed using un-paired two tailed t test, ***P <.001 (n=number of total embryos from three independent experiments) Scale bars: 200 µm (a-b-d); 100 µm (e).

Supplementary figure 4

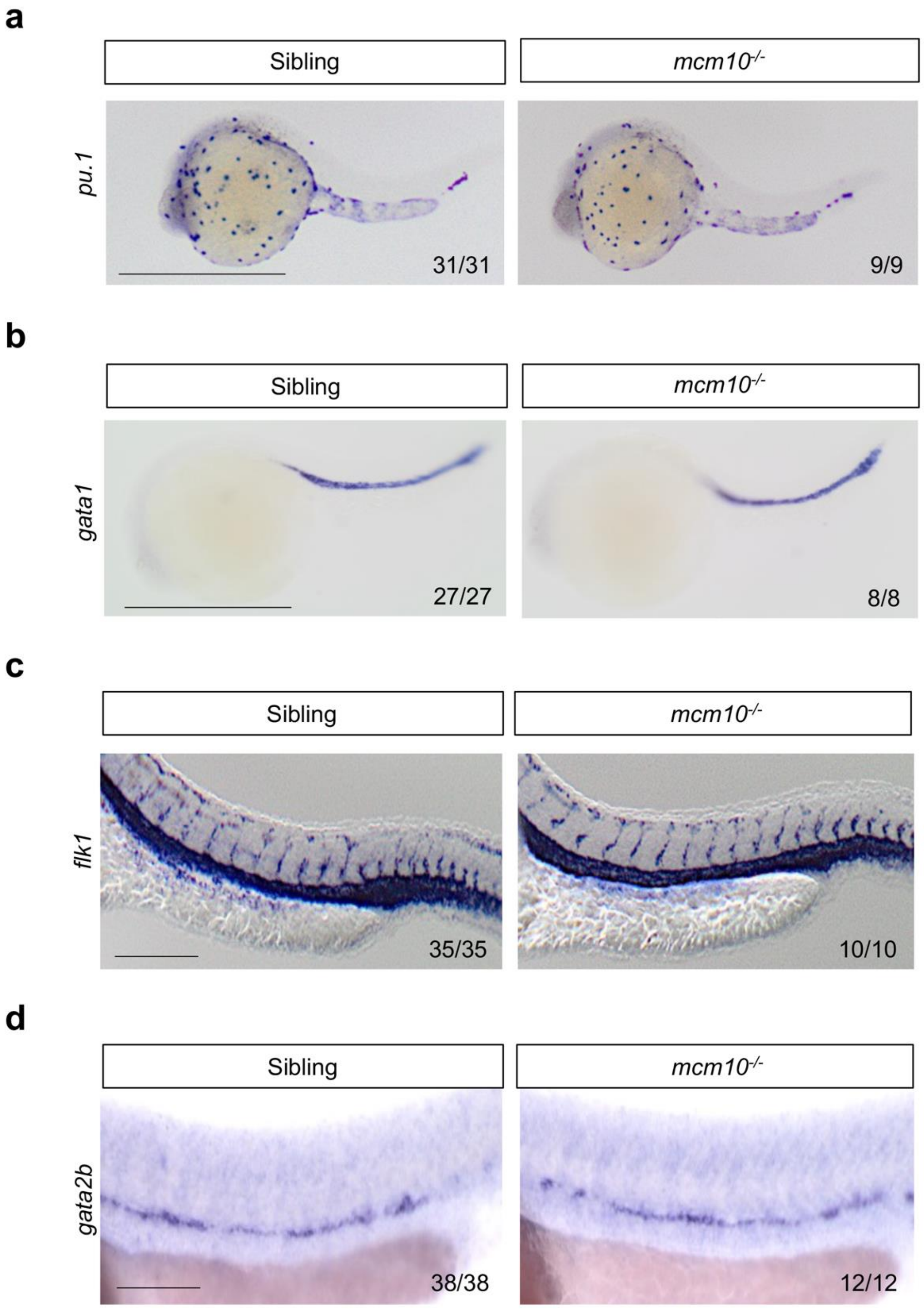

Suppl. Fig.4 The *mcm10* mutation does not affect primitive hematopoiesis or vasculogenesis. (Related to Figure 2).

WISH to detect the expression of: (a) *pu.1*; (b) *gata1*; (c) *flk1; (d) gata2b* in siblings and *mcm10*-mutant embryos. Scale bar: 200 µm (a-b), 100 µm (c-d).

Supplementary figure 5

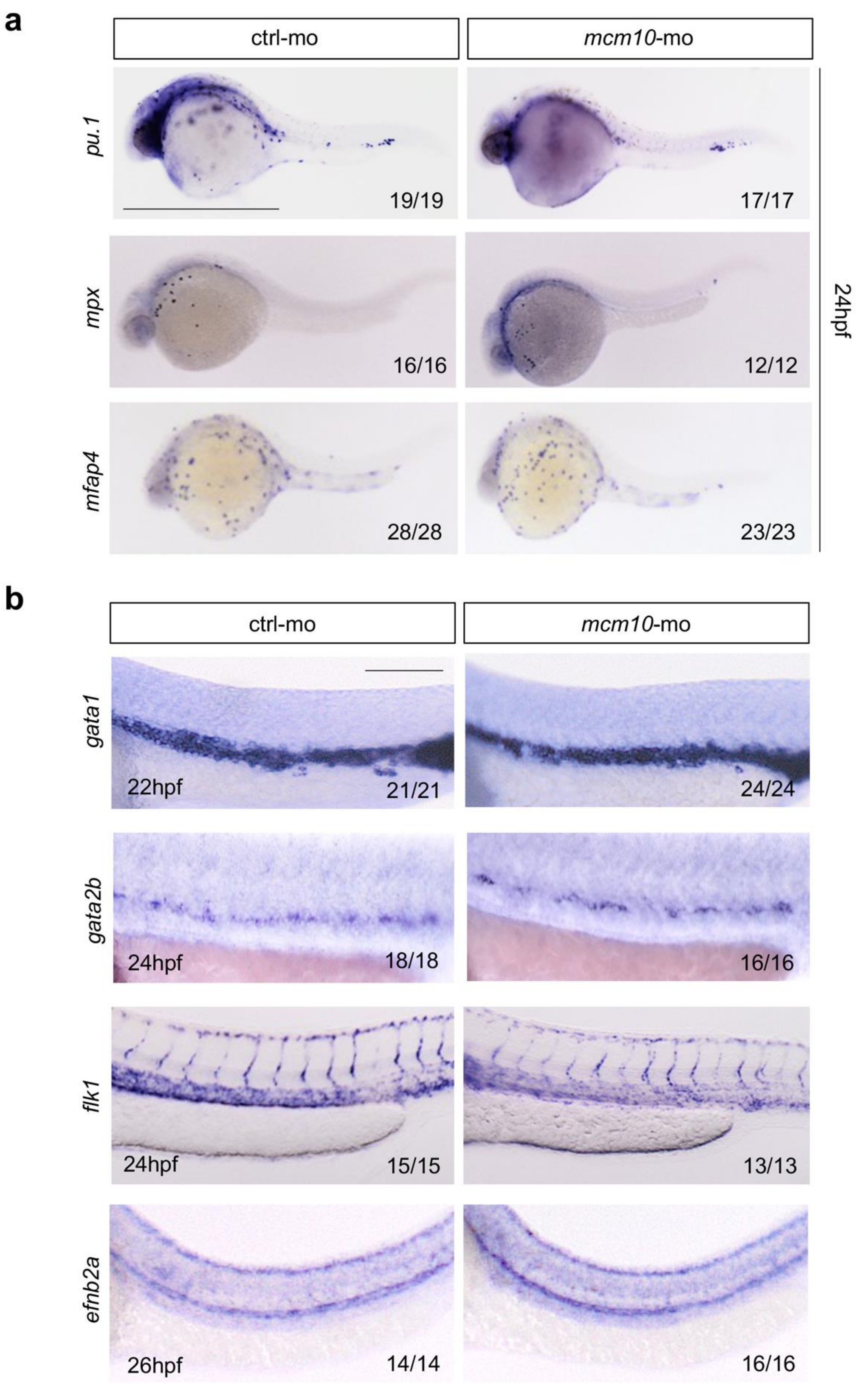

Suppl.Fig. 5 *Mcm10*-morphants have normal primitive hematopoiesis and vasculogenesis. (Related to Figure 2).

(a) WISH for *pu.1; mpx; mfap4* in 24hpf embryos injected with control- or *mcm10*-morpholino. *(b)* WISH for *gata1; gata2b; flk1 and efnb2a* in control- and *mcm10-morphants, performed at indicated stages*. Scale bars: 200 µm (a); 100 µm (b).

Supplementary figure 6

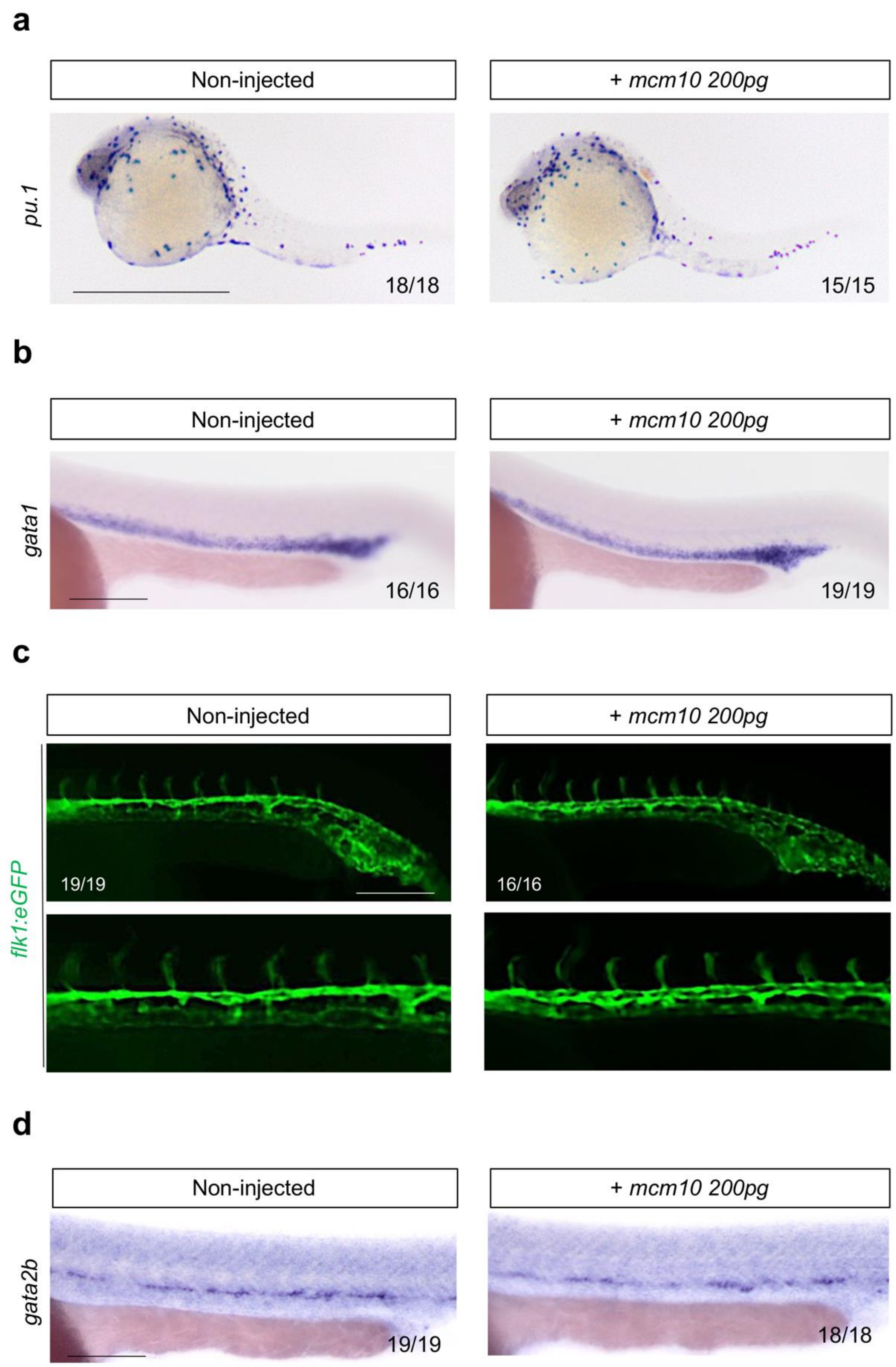

Suppl.Fig. 6 *Mcm10*-overexpression does not affect primitive hematopoiesis nor vasculogenesis. (Related to figure 4).

WISH to detect the expression of: (a) *pu.1*; (b) *gata1* in controls or siblings injected with mcm10 mRNA. (c) Fluorescence imaging of *flk1:eGFP* embryos either non-injected (controls) and injected with *mcm10* mRNA. (d) WISH to detect the expression of *gata2b.* Scale bars: 200 µm (a), 100 µm (b-c-d).

Supplementary figure 7

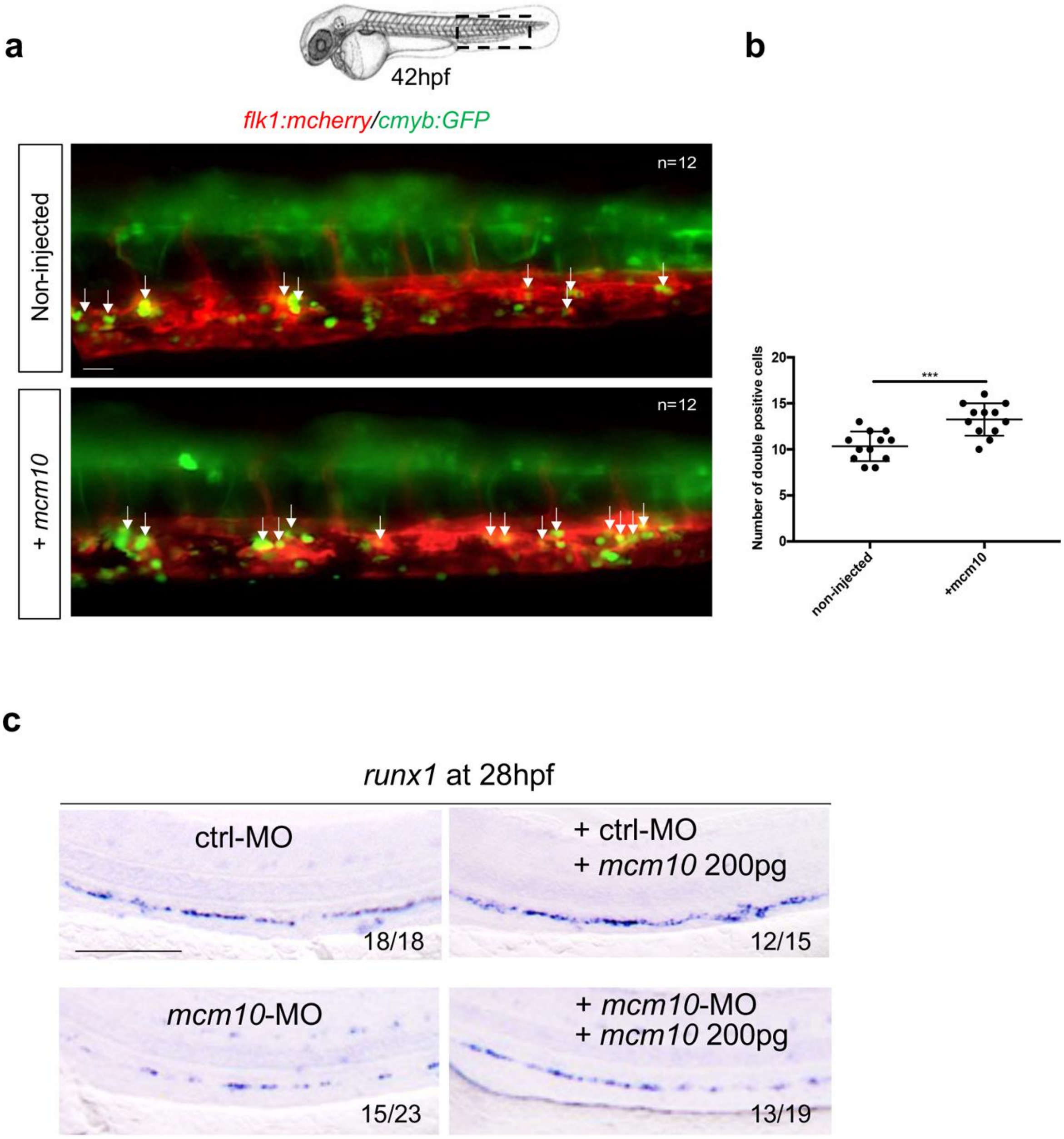

Suppl.Fig. 7 *Mcm10*-overexpression rescued the loss of HSPCs in mcm10-morphant. (Related to figure 5)

(a) Fluorescence imaging of *flk1:mcherry/cmyb:GFP* embryos non injected and injected with mcm10-RNA. (b) Centre values denote the mean, and error values denote s.e.m, statistical analysis was completed using un-paired two tailed t test, ***P <.001. (n=number of total embryos from three independent experiments). (c) WISH for *runx1* in embryo injected with control mcm10-morpholino and/or *mcm10-mRNA* Scale bars: 50 µm (a); 100 µm (c).
